# Sponsorship of oncology clinical trials in the United States according to age of eligibility

**DOI:** 10.1002/cam4.3083

**Published:** 2020-04-29

**Authors:** Dylan V. Neel, David S. Shulman, Clement Ma, Florence Bourgeois, Steven G. DuBois

**Affiliations:** ^1^ Harvard Medical School Boston MA USA; ^2^ Dana‐Farber/Boston Children's Cancer and Blood Disorders Center Harvard Medical School Boston MA USA; ^3^ Pediatric Therapeutics and Regulatory Science Initiative Computational Health Informatics Program Boston Children's Hospital Harvard Medical School Boston MA USA

**Keywords:** clinical trials, industry, oncology, pediatric, sponsorship

## Abstract

**Background:**

The sponsorship mix of trials relevant to young people with cancer has not been reported. Understanding this sponsorship mix may have implications for policies and regulations related to pediatric cancer drug development.

**Methods:**

We analyzed sponsorship of interventional trials first opened in the United States from 2007 to 2018 using the ClinicalTrials.gov registry. A total of 51 781 trials across non‐oncology disciplines and 18 431 oncology trials were classified according to lower age of eligibility (≥18 years vs < 18 years). Studies were stratified according to sponsorship (industry vs non‐industry). Trial characteristics were compared by sponsorship category. Trends in sponsorship were tracked over time.

**Results:**

Within oncology trials for patients ≥ 18 years, sponsorship was 33% industry and 67% non‐industry. Among oncology trials that included patients < 18 years, sponsorship was 16.6% industry and 83.4% non‐industry (*P* < .001). 15.5% of industry‐sponsored trials in non‐oncology disciplines included patients < 18 years, whereas only 5.2% of industry‐sponsored oncology trials were open to patients < 18 years (*P* < .001). Relative to trials with non‐industry sponsors, there was a statistically significant increase in industry sponsorship of oncology trials that included patients < 18 years over time (*P* < .001). Trial characteristics differed significantly according to sponsor type regardless of age of eligibility.

**Conclusions:**

Interventional oncology trials that include patients < 18 years are less likely to be industry‐sponsored compared to oncology trials exclusively in patients ≥ 18 years. Compared to other medical disciplines, a smaller proportion of industry‐sponsored oncology trials included patients < 18 years. Trial sponsorship is associated with differential trial characteristics, such as trial duration and number of patients enrolled, regardless of age.

## Graphical abstract

Trials relevant to young people with cancer in the United States are disproportionately sponsored by non‐industry sponsors. Oncology trials that include patients < 18 years are less likely to be industry‐sponsored compared to oncology trials for patients > 18 years, though rates of industry sponsorship have increased in recent years.

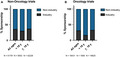

## 1 INTRODUCTION

The sponsor of a clinical trial is the single entity responsible for the overall conduct and oversight of the trial. Sponsors play a critical role in the design and reporting of clinical trials, with most trials sponsored by the pharmaceutical industry, government agencies, or academic institutions. Trial sponsorship has been shown to be associated with a number of factors, including likelihood of completion and publication of trial results.^1^, ^2^, ^3^, ^4^, ^5^

Prior studies have investigated the impact of clinical trial sponsorship on trial characteristics. These analyses suggest that elements of trial design, such as randomization, blinding, and use of data monitoring committees (DMCs), are influenced by sponsor type.^6^ For example, reported use of DMCs was less common in industry‐sponsored vs NIH‐sponsored trials, likely due to NIH mandates for DMCs for government‐funded trials.^6^ Several studies have also suggested that trial outcomes are associated with sponsorship status.^7^, ^8^, ^9^ Industry‐led trials are more likely to have positive trial outcomes compared to NIH or academic‐led trials, with one analysis reporting that industry sponsorship was also associated with decreased reporting of results.^4^ There have been more recent studies investigating temporal trends in trial sponsorship within the United States. In an analysis of trials registered on ClinicalTrials.gov, one group reported a decrease in trials sponsored or funded by the NIH from 2006 to 2014.^10^

Data on sponsorship of pediatric clinical trials are sparse. In one analysis, 6.7% of trials either sponsored or funded by the NIH were exclusively focused on pediatric populations, with another 7% of trials open to adults and children.^11^ In another analysis of pediatric interventional clinical trials across disciplines, 32.3% were industry‐sponsored, 7.8% were government‐sponsored, and 59.9% were sponsored by academic institutions or other organizations.^12^ Little work has investigated sponsorship status specifically in trials relevant to children with cancer. In this context, we sought to examine the distribution of sponsor types for oncology trials relevant to young people. We compared trial sponsorship according to age of eligibility for oncology trials and for trials in other disciplines. We examined whether sponsorship is associated with differences in trial characteristics (eg phase, duration, enrollment). Understanding the current landscape of sponsorship of trials relevant to young people with cancer may have important implications for advocacy and policy efforts to accelerate cancer drug development for this population.

## 2 METHODS

### 2.1 Trial search and variables

We used the ClinicalTrials.gov registry to evaluate interventional trials with at least one US participating site with trial start date from September 27, 2007 (start of US mandatory trial registration) to December 1, 2018. Interventional trials were defined according to the NIH/NCI definitions that mirror the definition used in ClinicalTrials.gov, and included both interventional treatment (for the primary disease) and interventional non‐treatment (for a secondary condition). Data for trials across all disciplines were included and divided into oncology trials and trials of other disciplines using the disease under study from the search function within ClinicalTrials.gov. Sponsorship was analyzed categorically as “industry” or “non‐industry.” Industry sponsors included any commercial entity, including pharmaceutical, biotech, and device companies. Non‐industry sponsors included academic, government, community organizations, or non‐academically affiliated hospitals. Information on available trial characteristics as follows was extracted from trial records. Trial duration (end date minus start date for completed trials), number of patients enrolled (for completed trials), trial phase (I‐IV), trial status, and results reporting in ClinicalTrials.gov (yes/no) were assessed from data in the registry as of December 2, 2018. Completed trials were coded as having results reported if any study results were provided to the registry to be posted on ClinicalTrials.gov. Trials were classified according to lower age of eligibility (trials exclusively open to enrolling patients ≥ 18 years vs trials that included patients < 18 years even if eligibility included older patients). Trial data were downloaded directly from ClinicalTrials.gov.

### 2.2 Statistical analysis

Chi‐squared tests were used to compare proportions between non‐ordered groups. *T* tests and Wilcoxon‐rank sum tests were used to compare continuous data between normally and non‐normally distributed groups, respectively. Logistic regression in R version 3.5.0 was used to evaluate potential changes in sponsorship mix over time, using the larger sponsorship group (non‐industry sponsors) as the reference group. All other statistical analyses were performed in Stata.

## 3 RESULTS

### 3.1 Trial search and overall sponsorship

**Figure 1:**
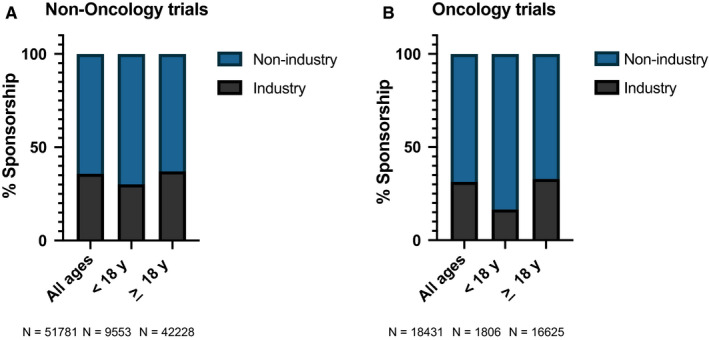
Sponsorship mix of US interventional clinical trials stratified by age of eligibility for all non‐oncology trials (n = 51 781; panel A) and interventional oncology trials (n = 18 431; panel B)

We identified 70 413 interventional trials across all disciplines, of which 51 955 were interventional non‐oncology trials and 18 458 were interventional oncology trials. Of these, 51 781 (99.7%) non‐oncology trials and 18 431 (99.8%) oncology trials had sponsorship data available. Across all non‐oncology disciplines, trial sponsorship was 35.8% industry and 64.2% non‐industry (Figure 1A). Among all interventional oncology trials, sponsorship mix was 31.3% industry and 68.7% non‐industry (Figure 1B).

### 3.2 Sponsorship mix according to age of trial eligibility

We next stratified trials by age of eligibility and analyzed for sponsorship distribution. Within 42 228 non‐oncology interventional trials (for which sponsorship data were available) for patients ≥ 18 years, 37.1% were sponsored by industry and 63.0% by non‐industry (Figure 1A). The 9,553 non‐oncology trials for which patients < 18 years were eligible were less likely to be industry‐sponsored (30.1%) relative to trials for patients ≥ 18 years (*P* < .001). This pattern was exacerbated within interventional oncology trials (Figure 1B). Specifically, 33.0% percent of 16 625 oncology trials for patients ≥ 18 years were industry‐sponsored, compared to 16.6% of 1,806 studies for which patients < 18 years were eligible (*P* < .001). Figure S1 shows these data presented as absolute number of trials in each category and demonstrates the paucity of industry‐sponsored oncology trials that include patients < 18 years. As non‐industry sponsors comprised both government and non‐government sponsors, a breakdown of sponsorship by industry vs government vs non‐government/non‐industry is shown in Figure S2.

We next investigated potential differences in proportion of industry trials that included patients < 18 years between non‐oncology and oncology trials. In a sub‐analysis of the 18 520 industry‐sponsored non‐oncology trials, 15.5% of these trials allowed patients < 18 years. Among the 5,778 industry‐sponsored oncology studies, only 5.2% of trials allowed patients < 18 years (*P* < .001).

**Figure 2:**
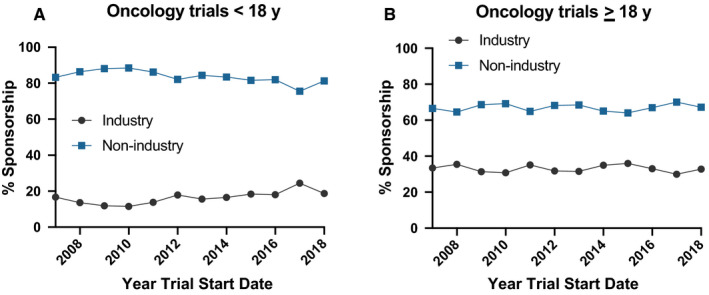
Sponsorship mix of US interventional oncology trials that inlclude patients < 18 years (panel A; n=1806 across all years) and for trials for patients >= 18 years (panel B; n=16 625 across all years)

We next evaluated temporal trends (2007‐2018) in sponsorship mix of newly opened trials over time (Figure 2). For oncology trials for which patients < 18 years were eligible (Figure 2A), we observed consistently high sponsorship by non‐industry sponsors over time. There was a significant increase in industry sponsorship compared to the reference group of non‐industry‐sponsored trials over time (odds ratio for being industry‐sponsored of 1.07; 95% confidence interval 1.03‐1.11; *P* < .001) In other words, there was a 7% increase in odds of a trial having an industry vs non‐industry sponsor for every one‐year increase in trial start date. There was no change in proportion of oncology trials for patients ≥ 18 years sponsored by industry compared to non‐industry over time (odds ratio 0.997; 95% confidence interval 0.99‐1.01; *P* = .52). Figure S3 shows these data presented as absolute number of trials in each category.

### 3.3 Impact of sponsorship on trial characteristics

We next evaluated trial characteristics according to sponsor type (Table 1). For oncology trials for patients ≥ 18 years, industry sponsored trials were more likely to be phase I or III, and less likely to be phase II than non‐industry trials. For trials that included patients < 18 years, industry trials were more likely to be phase I/II or III, and less likely to be phase I or II, than other trials. Regardless of age of eligibility, industry‐sponsored trials had shorter durations and larger median enrollments. Non‐industry‐sponsored oncology trials for patients ≥ 18 years had slightly higher termination rates compared to industry trials (18.1% vs 16.9%; *P* < .001). Results were more likely to be reported to the registry for industry‐sponsored trials, particularly for oncology trials that allowed patients < 18 years.

**Table 1:** Characteristics of oncology interventional clinical trials according to sponsor type and age of eligibility

Characteristic	All oncology trials^b^ (n = 18 431)	Trials that only include patients ≥ 18 y (n = 16 625)			Trials that include patients < 18 y (n = 1806)		
		Industry Sponsored (n = 5479)	Non‐Industry Sponsors (n = 11 146)	*P*‐value	Industry sponsored (n = 299)	Non‐industry sponsors (n = 1507)	*P*‐value
Trial phase (%)^b^				<.001			<.001
Phase I	34.7	38.7	32.1		24.2	38.4	
Phase I/II	13.4	14.7	12.5		19.9	11.4	
Phase II	40.5	28.5	48.4		35.7	41.1	
Phase III	9.6	16.9	4.9		18.1	7.3	
Phase IV	1.8	1.3	2.1		2.2	1.8	
Mean Trial Duration (y)^c^ (*SD*)	3.7 (2.2)	3.3 (2.0)	4.0 (2.3)	<.001	3.4 (2.1)	4.5 (2.4)	<.001
Median enrollment (n)^d^(Interquartile range)	36 (13‐100.5)	100 (36‐278)	24 (10‐53)	<.001	43 (22‐114.5)	28.5 (10.5‐67)	.002
Trial status (%)^e^				<.001			.32
Recruiting	30.8	23.0	33.5		31.3	38.9	
Not recruiting	0.1	0.1	0.2		0.0	0.1	
Completed	46.9	57.4	42.5		47.2	40.6	
Terminated	17.5	16.9	18.1		16.7	15.6	
Withdrawn	4.0	2.3	5.0		4.1	3.7	
Enroll by invitation	0.7	0.4	0.8		0.8	1.1	
Results Reported (%)				<.001			.005
Yes	24.1	25.8	23.3		28.1	20.7	

^a^ Variables with proportional data show proportions sum within columns.

^b^ Data for trial phase available in 14 731 trials.

^c^ Trial duration calculated as time from study start to study completion date only for completed trials with available duration data (n = 6736).

^d^ Trial enrollment calculated only for completed trials with results reported (n = 4839).

^e^ Data for trial status available in 14 428 trials.

## 4 DISCUSSION

These results provide a comprehensive view of the sponsorship mix of interventional oncology trials available to patients < 18 years in the United States. Sponsorship distribution can have implications for policies seeking to stimulate new drug development within specific populations of patients, including pediatrics. We found that sponsorship mix of oncology trials for patients ≥ 18 years parallels sponsorship mix in other disciplines. In contrast, oncology trials that include patients < 18 years were less likely to be industry‐sponsored compared with oncology trials for patients ≥ 18 years and compared with trials in other disciplines that included patients < 18 years. This finding is significant as we observed that industry‐sponsored trials were more likely to be phase III trials, tended to enroll more patients, but required less time to do so. We did note higher odds of oncology trials that included patients < 18 years to be industry‐sponsored in more recent years.

A key finding from this analysis is that industry‐sponsored oncology trials focused disproportionately on adults. In a prior analysis, 32.3% of pediatric trials across disciplines were industry‐sponsored,^12^ compared to 16.6% for oncology trials that included patients < 18 years in our analysis. Recent US legislation to hasten pediatric cancer drug development (eg RACE for Children Act) and efforts to lower the age of eligibility for oncology trials may increase industry sponsorship of pediatric oncology trials,^13^, ^14^, ^15^ and potentially bring novel agents to children with cancer earlier.^16^

A previous analysis observed a marked increase in industry sponsorship of randomized trials of common adult cancers from 1975 to 2004 in the United States (4% to 57%).^17^ Our current findings complement these results by demonstrating that the increase in industry sponsorship over time extends to oncology trials that include patients < 18 years of age as well. Comparative data on oncology sponsorship mix from other countries are limited, with one study showing 43% industry sponsorship in Australia compared to our finding of 31%.^18^

Nearly half of oncology trials with non‐industry sponsors in both adults and children were phase II trials, which may reflect challenges of conducting first‐in‐human trials and large randomized trials for sponsors other than industry. This metric will be of interest to follow over time in the era of molecularly targeted agents that may obtain regulatory approval based upon high response rates in phase II trials.^19^ Given this shifting paradigm, it is possible that the proportion of industry‐sponsored phase II trials will increase with time in both adults and children. It is also noteworthy that industry‐sponsored oncology trials that included patients < 18 years were enriched for phase I/II trials compared to trials from other sponsors. This finding may reflect a greater willingness to commit to a single phase I/II trial of an agent that has already completed adult phase I testing or a desire to use more efficient strategies for pediatric development.

We acknowledge certain limitations in this analysis. Sponsorship should not be conflated with trial funding since the sponsor does not necessarily provide the funds to conduct the trial. For example, a trial sponsored by an academic institution may be funded by a federal grant, with drug supplied by an industry collaborator. Support for pediatric oncology trials in the United States is particularly complex as the NCI‐funded Children's Oncology Group (COG) is a common trial sponsor, though some trials receive supplemental financial support from industry. ClinicalTrials.gov lacks granular data on trial funding, though this topic would be of interest in subsequent analyses. Our analysis provides new descriptive data, but as a registry‐based study, we are unable to provide definitive explanations for observed trends and differences. Furthermore, due to a lack of other comprehensive registries providing sponsorship information for trials in the United States, we were unable to validate our findings by comparing with independent sources. Given the data available in ClinicalTrials.gov, we made an a priori decision to focus only on trials open in the United States, though future analyses may focus on this issue more globally. Likewise, it is likely that some of the trials that allowed patients < 18 years to participate ultimately enrolled no children. Moreover the structure of the data in ClinicalTrials.gov precluded an analysis that separated trials based upon age categories below age 18 years (eg trials allowing patients 12 years and older vs trials that included patients younger than 12 years). Finally, with only 299 industry‐sponsored oncology trials that included patients < 18 years, some sample sizes based upon specific trial characteristics were limited.

In summary, we provide a comprehensive analysis of sponsorship of interventional oncology trials available to patients < 18 years in the modern era. As pediatric cancer drug development paradigms and regulations evolve over time, it will be important to re‐assess these findings.

## Supporting information

Fig S1‐S3
