# Supplementary material for: Sponsorship of oncology clinical trials in the United States according to age of eligibility
Source: Cancer Med. 2020 Apr 29;9(13):4495–500. doi: 10.1002/cam4.3083 (PMC7333829; doi:10.1002/cam4.3083)
Supplement: Supplementary file 1 — Fig S1‐S3 [file CAM4-9-4495-s001.pptx]

## Slide 1
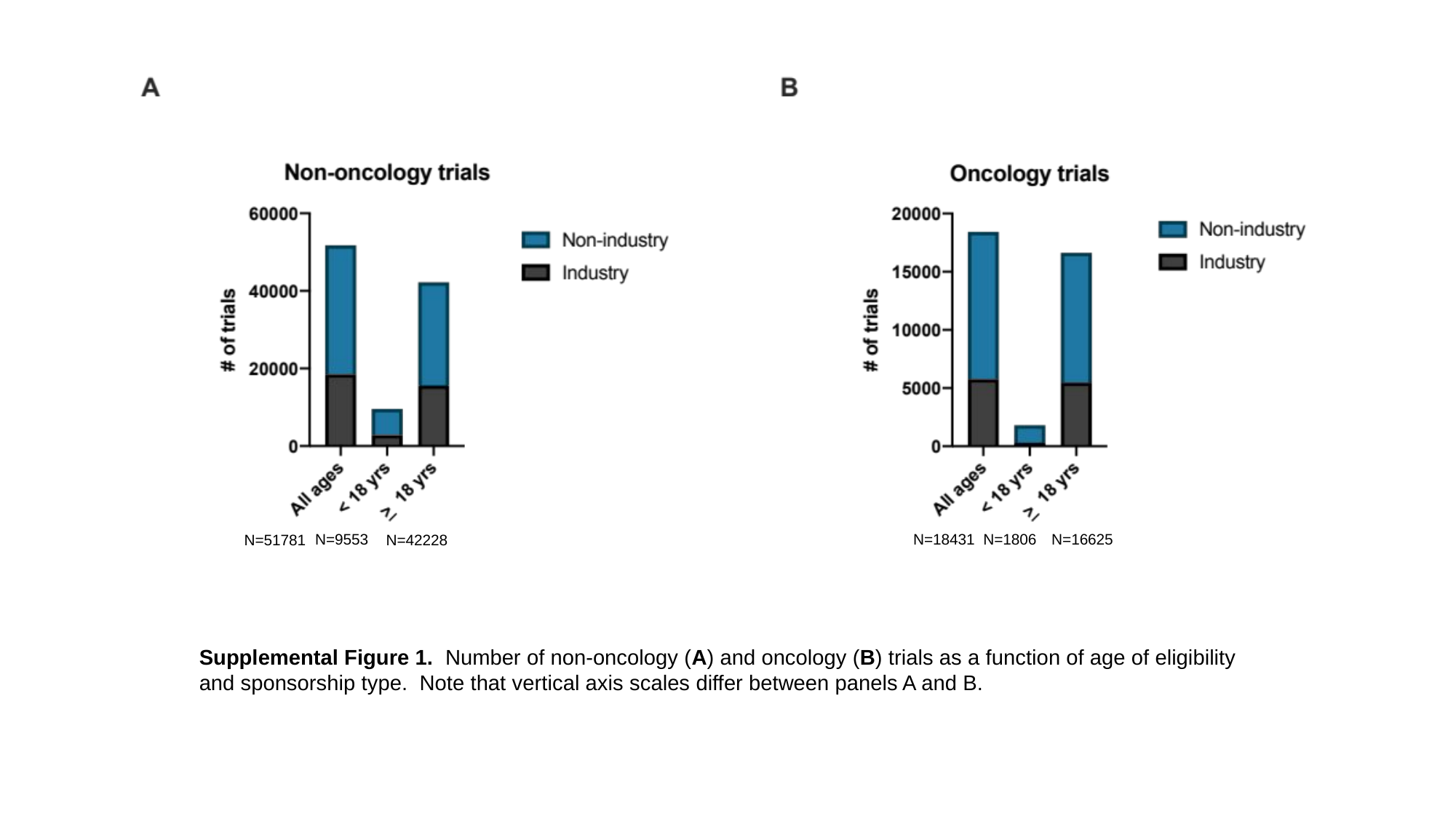

N=18431
N=1806
N=16625
N=9553
N=42228
N=51781
Supplemental Figure 1. Number of non-oncology (A) and oncology (B) trials as a function of age of eligibility
and sponsorship type. Note that vertical axis scales differ between panels A and B.

## Slide 2
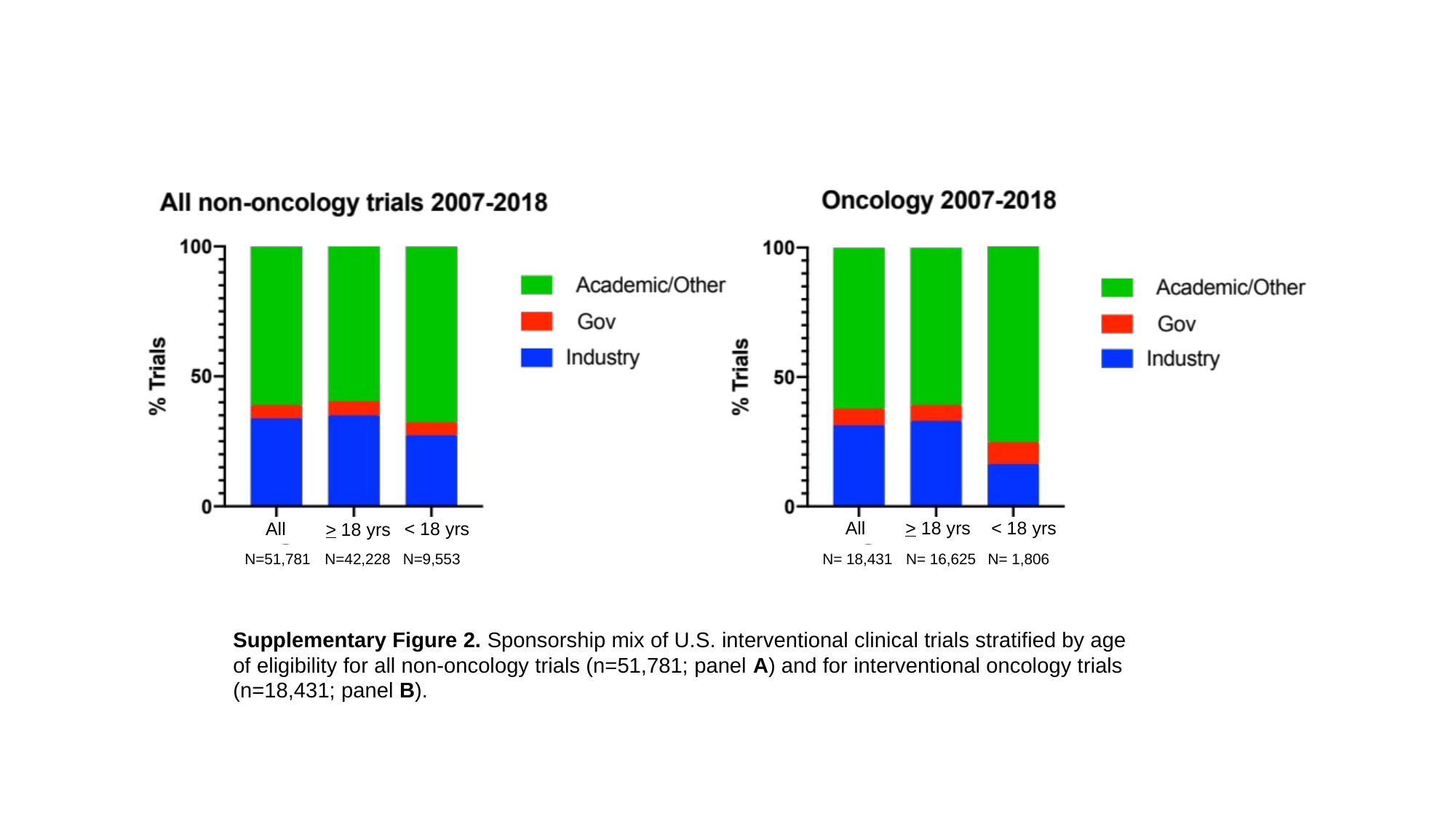

All
< 18 yrs
> 18 yrs
All
< 18 yrs
> 18 yrs
N=51,781
N=42,228
N=9,553
N= 18,431
N= 16,625
N= 1,806
Supplementary Figure 2. Sponsorship mix of U.S. interventional clinical trials stratified by age of eligibility for all non-oncology trials (n=51,781; panel A) and for interventional oncology trials (n=18,431; panel B).

## Slide 3
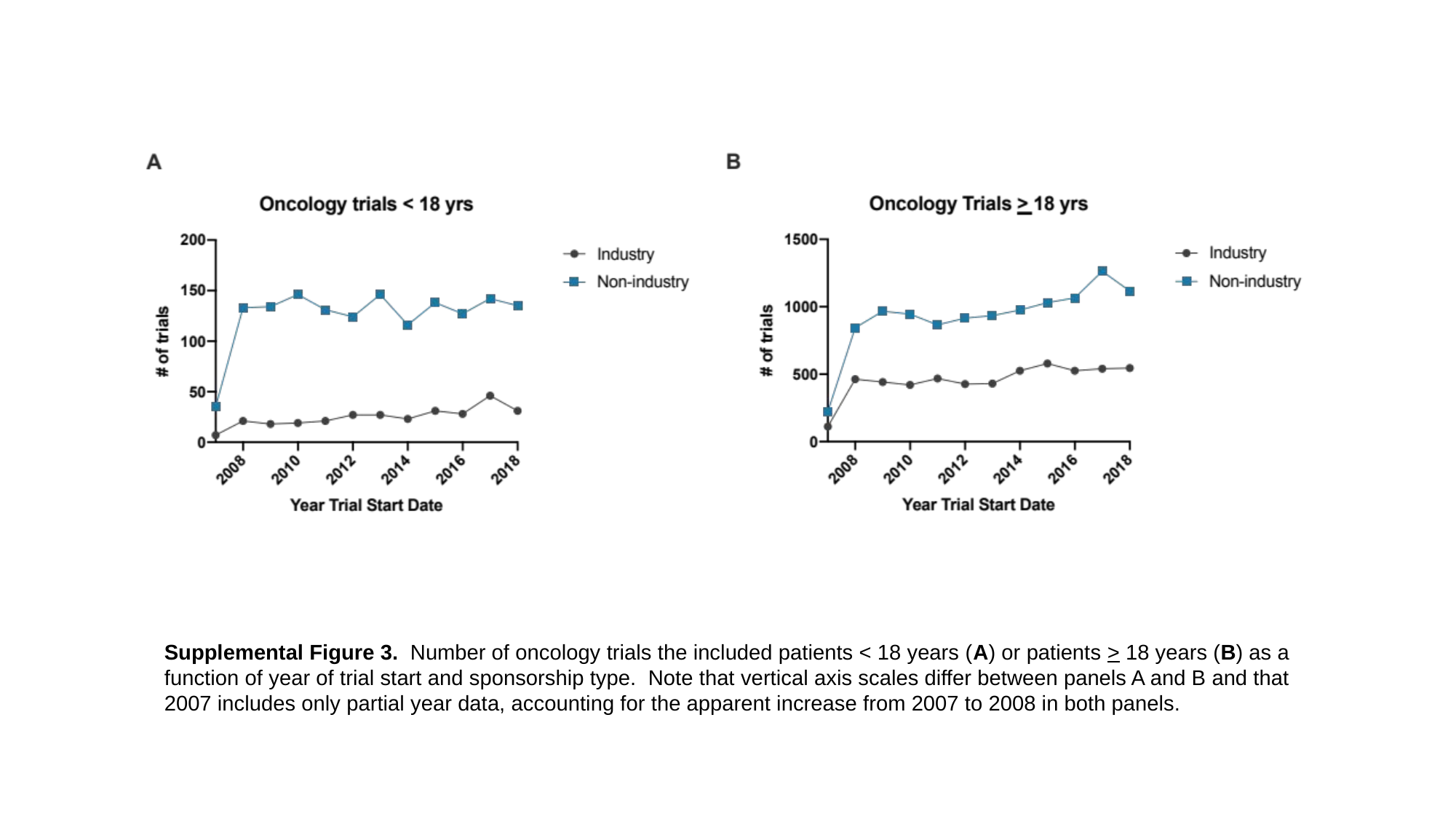

Supplemental Figure 3. Number of oncology trials the included patients < 18 years (A) or patients > 18 years (B) as a
function of year of trial start and sponsorship type. Note that vertical axis scales differ between panels A and B and that
2007 includes only partial year data, accounting for the apparent increase from 2007 to 2008 in both panels.
